# Integrating network pharmacology, bioinformatics, and experimental validation to unveil the molecular targets and mechanisms of galangin for treating hepatocellular carcinoma

**DOI:** 10.1186/s12906-024-04518-x

**Published:** 2024-05-30

**Authors:** Xiaoliang Li, Mingyan Zhou, Weijia Chen, Jiangbo Sun, Yihang Zhao, Gaoan Wang, Bingshu Wang, Yipeng Pan, Junqing Zhang, Jian Xu

**Affiliations:** 1 Hepatobiliary and Liver Transplantation Department of Hainan Digestive Disease Center, Institute of Clinical Medicine, The Second Affiliated Hospital of Hainan Medical University, No. 368 Yehai Road, Haikou, Hainan Province 570311 China; 2 Engineering Research Center of Tropical Medicine Innovation and Transformation of Ministry of Education & International Joint Research Center of Human-Machine Intelligent Collaborative for Tumor Precision Diagnosis and Treatment of Hainan Province & Hainan Provincial Key Laboratory of Research and Development on Tropical Herbs & Haikou Key Laboratory of Li Nationality Medicine, School of Pharmacy, Hainan Medical University, No. 3 Xueyuan Road, Haikou, 571199 Hainan Province China

**Keywords:** Galangin, Hepatocellular carcinoma, Network pharmacology, Bioinformatics, Molecular docking

## Abstract

**Background:**

Galangin, a flavonoid compound, is derived from *Alpinia officinarum* Hance. Previous studies have shown that galangin can inhibit the proliferation of hepatocellular carcinoma (HCC), but its mechanism is still unclear. This study aims to investigate the potential targets and molecular mechanisms of galangin on HCC through network pharmacology, bioinformatics, molecular docking, and experimental in vitro validation.

**Methods:**

In this study, network pharmacology was used to investigate the targets and mechanisms of galangin in the treatment of HCC. AutoDockTools software was used to simulate and calculate the binding of galangin to its core targets. GO and KEGG enrichment analyses were conducted in the DAVID database to explore the main biological functions and signaling pathways impacted by galangin intervention. In addition, bioinformatics was applied to examine the correlation between the differential expressions of the anti-HCC core targets of galangin and the survival of patients with HCC. Finally, the findings obtained from network pharmacology and bioinformatics were verified in cell experiments.

**Results:**

A total of 67 overlapping target genes of galangin and HCC were identified. Through the analysis of the protein-protein interaction (PPI) network, 10 hub genes with the highest degree of freedom were identified, including SRC, ESR1, MMP9, CDK4, CCNB1, MMP2, CDK2, CDK1, CHK1, and PLK1. These genes were found to be closely related to the degradation of the extracellular matrix, signal transduction, and the cell cycle. GO and KEGG enrichment analyses revealed that galangin exerts an anti-HCC role by affecting various signaling pathways, including the cell cycle, pathways in cancer, and the PI3K-Akt signaling pathway. The results of molecular docking indicated a significant interaction between galangin and CCNB1, CDK4, CDK1, and PLK1. Bioinformatics analysis revealed that CCNB1, CDK4, CDK1, and PLK1 were upregulated in the liver of patients with HCC at both the mRNA and protein levels. Flow cytometry analysis showed that galangin induced G0/G1 phase arrest and cell apoptosis in HepG2 and Huh7 cells. Additionally, galangin suppressed the expression of key proteins and mRNAs involved in the cell cycle pathway.

**Conclusions:**

These results suggest that galangin inhibits the growth of HCC cells by arresting the cell cycle at the G0/G1 phase.

**Supplementary Information:**

The online version contains supplementary material available at 10.1186/s12906-024-04518-x.

## Introduction

Hepatocellular carcinoma (HCC) is the most common type of liver cancer. Patients diagnosed with early-stage HCC, who have had the tumor completely removed, can anticipate positive treatment outcomes. Nevertheless, the early symptoms of HCC are not easily noticeable, and when it is eventually diagnosed, it is usually in the late stage. Patients suffering from advanced HCC, who have missed the optimal period for surgical treatment, are often only able to be treated with drugs. Despite its efficacy in symptom management and disease control, sorafenib, the first-line drug for advanced HCC, often triggers serious adverse reactions such as diarrhea, gastrointestinal bleeding, and hypertension during long-term use [1]. Therefore, it is the focus of current research to continuously explore therapeutic targets for HCC and develop highly efficient and specific targeted drugs for the treatment of HCC.

Evidence has shown that traditional Chinese medicine can be beneficial in alleviating symptoms and enhancing the quality of life of patients with HCC, inhibiting the recurrence of the tumor, and controlling the progression of the disease [2]. Galangin, a flavonoid derived from nature, is present in *Alpinia officinarum* Hance, *Plantago depressa* Willd., and propolis [3]. Numerous studies [4–6] have revealed that galangin has a comprehensive range of anti-tumor effects, including colon cancer, breast cancer, leukemia, lung cancer, esophageal cancer, and HCC. Galangin can regulate the expression of cyclin D1, CDK4, CDK6, and other cell cycle-related proteins, inhibit the transformation of colon cancer cells from the G0/G1 phase to the S1 phase, and ultimately induce cell cycle arrest in cancer cells [7]. Galangin can also affect the expression of cyclin D3, cyclin E, and cyclin A, which causes human breast cancer cells to be blocked in the G0/G1 phase [8]. In addition, galangin may exert a potent inhibitory effect on diethylnitrosamine (DEN)-induced HCC by suppressing the PI3K/AKT signaling pathway [5]. It is capable of triggering apoptosis in HepG2 hepatoma cells through the inhibition of the cell cycle, reduction of the mitochondrial membrane potential, and disruption of intracellular calcium ion homeostasis [9]. However, the mechanism by which galangin inhibits HCC by regulating specific proteins in the cell cycle remains to be elucidated.

In the present study, network pharmacology was first used to predict the potential targets and signaling pathways of galangin in the treatment of HCC. Subsequently, molecular docking techniques were used to evaluate the binding affinity of galangin with the core targets. Bioinformatics was used to analyze the differential expression of the core targets in HCC. To elucidate the potential mechanism of galangin in the treatment of HCC, HepG2 and Huh7 cells were used in the study. This study has unveiled a novel mechanism of action of galangin in the treatment of HCC and provided a new idea and theoretical basis for treating HCC with small-molecule compounds derived from traditional Chinese medicine.

## Materials and methods

### Prediction of potential targets for the treatment of HCC with galangin

The predicted targets of galangin from the SwissTargetPrediction website were intersected with the targets of galangin screened from the BATMAN-TCM database. After being corrected by the UniProt Knowledgebase, the drug targets of galangin were determined. The keyword “Hepatocarcinoma” was used to search the databases, including PharmGKB, DisGeNET, OMIM, and GeneCards, with the search limited to the species “Homo sapiens”. Subsequently, the disease targets of HCC were gathered by applying screening conditions of a GeneCards score of ≥ 15 and a DisGeNET value of ≥ 0.10. Duplicate entries were then removed. The drug target of galangin and the disease target of HCC were intersected using Venny2.1 to obtain the anti-HCC target of galangin.

### Construction of the intersection network and PPI network

By setting the species as “Homo sapiens” and utilizing a confidence level of 0.4, STRING11.5 was accessed to construct a protein-protein interaction (PPI) network map to explore the targets of galangin in the treatment of HCC. The core targets in the “galangin-target-pathway-HCC” network were identified through the analysis of key nodes in the PPI network using Cytoscape 3.9.1 software, based on their degree centrality values.

### Gene ontology (GO) and kyoto encyclopedia of genes and genomes (KEGG) enrichment analyses

By uploading the galangin target to the DAVID database, specifying the species as “Homo sapiens” and setting the threshold at *p* < 0.05, GO function and KEGG pathway enrichment analyses can be conducted.

### Molecular docking

The molecular structure of galangin was obtained from the PubChem database. The molecular structure of the selected core target protein was downloaded from the PDB database and imported into PyMOL 2.4.0 software. Water molecules and residues were removed using software. The core target protein structure that had been processed was used as the receptor, while the molecular structure of galangin served as the ligand. The information was then uploaded to AutoDock Tools for further calculations and docking simulations. The docking data was finally visualized and analyzed using the PyMOL software.

### Bioinformatics analysis of the differential expression of core targets in HCC

The transcriptome data for the core genes selected in this study was obtained from the TCGA database. The mRNA expression differences of the core targets in human HCC tissues were analyzed using the R language. The CPTAC database was used to download protein data for core targets and analyze their protein expression differences in HCC tissues using the R language. The Kaplan-Meier plotter database was used to investigate the relationship between the expression of core target genes and the survival of HCC patients.

### Cell culture and treatment

The human hepatoma cell lines HepG2 and Huh7, as well as the human hepatic stellate cell line LX-2, were purchased from the Bena Culture Collection (BNCC, Suzhou, China). HepG2 cells were cultured in DMEM medium, Huh7 cells in RPMI 1640, and LX-2 cells in RPMI 1640. All media were supplemented with 10% FBS (v/v) and 1% penicillin-streptomycin, and were kept in a humid atmosphere with 5% CO_2_ at 37 °C.

### Cell viability assay

A density of 1 × 10^4^ cells per well was seeded into 96-well plates, with 5 replicates per group, and incubated at 37 °C and 5% CO_2_ for 12 h. After 12 h of medication, the liquid in the 96-well plates was discarded. Next, all wells were supplemented with 100 µL of medium containing CCK8 and incubated at 37℃ and 5% CO_2_ for 1 h. After incubation, the absorbance was measured at 450 nm using an enzyme marker.

### Cell proliferation assays

Cells in the logarithmic growth stage were exposed to galangin at concentrations of 0, 40, 80, and 120 µM. Following a 12 h incubation period, each well was exposed to a 2x EdU working solution at 37℃ and 5% CO_2_. Then, the cells were fixed with 4% paraformaldehyde for 15 min and permeabilized with 0.25%Triton X-100 for 15 min. The cells were incubated with the Click Additive Solution for 30 min and Hoechst for 10 min, respectively, and then observed under a fluorescence microscope.

### Cell apoptosis and cell cycle analysis

Cells from various treatment groups were transferred into clean flow tubes, and Annexin V-FITC and PI staining were added to each tube, respectively, according to the instructions of the apoptosis kit. The tubes were left in darkness for 10–15 min before the cells were combined with Annexin V 1x Binding Buffer and placed on ice. The apoptosis rate of each group was determined using flow cytometry. Cells from different treatment groups were fixed with 80% ethanol, and the PI staining solution was added according to the instructions of the cell cycle kit.

### Western blotting analysis

Cell samples from different treatment groups were lysed in cell lysate, and the protein concentrations were determined according to the procedure of the BCA kit. The same amount of protein extract was subjected to 10% SDS-PAGE and then transferred to a PVDF membrane. The membrane with the protein was enclosed in 5% skim milk powder. After washing, the membranes were left to incubate with primary antibodies at 4 ℃ overnight. The next day, after washing the membrane, the second antibody was incubated at room temperature. After ECL color development, an imaging system with high sensitivity for chemiluminescence was used for exposure development. The band intensity was quantified using Image Lab image processing software.

### Quantitative real-time PCR (qPCR)

The total RNA from the cells was extracted using Trizol reagent, and its concentration was determined using NanoDrop One. Then, the total RNA was reverse transcribed into cDNA and amplified by PCR. The mRNA level was normalized to the endogenous housekeeping gene GAPDH and was expressed relative to the calibrator sample using the 2 ^−ΔΔCt^ method. The analyzed genes and their corresponding primer sequences are listed in Table 1.

**Table 1:** Primer sequences

Gene	Sequence (5’-3’)	
ATR	F: TTGTCTGGGCTCCCTTC	R: GCACAGCATCCCTGTTTT
CHK1	F: GTCACAGGAGAGAAGGCAA	R: AGCATCTGGTTCAGGCA
CDC25C	F: CGAGCGGGGATAGGTTA	R: CTGAGCAGAAGGCCAAAG
CCNB1	F: CTTTCGCCTGAGCCTATTT	R: CCATCTTCTGCATCCACAT
CDK4	F: AAGGAAGAAAAGCTGCCAT	R: TCAAAAGGAGAGGTGGGA
CDK1	F: AAAGTGAAGAGGAAGGGGTT	R: TGTACTGACCAGGAGGGATAG
PLK1	F: TGCACCGAAACCGAGTTA	R: CCACACAGGGTCTTCTTCC
GAPDH	F: GGGGCTCTCCAGAACATC	R: TGACACGTTGGCAGTGG

### Statistical analysis

All the experimental data were expressed as mean ± SD. Statistical analyses were conducted using GraphPad Prism 9.0. Data conforming to normal distribution were analyzed by one-way ANOVA. The results of the homogeneity of variance were compared between groups using the LSD test. Dunnett’s T3 test was used for unequal variances.

## Results

### Network pharmacology analysis of galangin

**Fig. 1:**
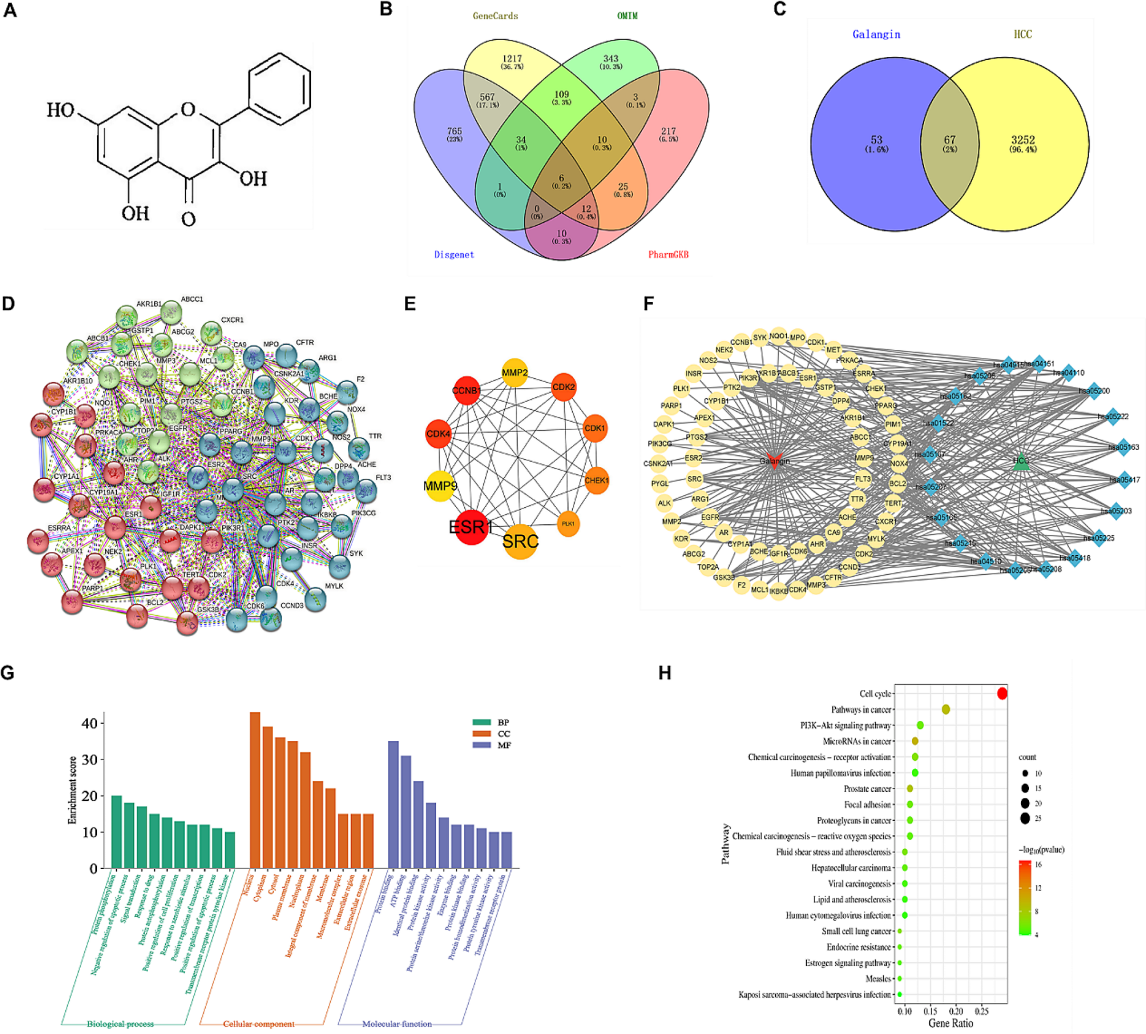
Network pharmacology predicted the potential mechanisms of galangin for HCC. (**A**) The chemical structure formula of galangin. (**B**) The Venn diagram of the HCC-related targets in the GeneCards, OMIM, DisGeNET, and PharmGKB databases. (**C**) The Venn diagram of the targets shared by galangin and HCC. (**D**) The top ten core targets of degree of freedom values in topological analysis. (**E**) PPI network. (**F**) Network diagram of galangin-pathway-HCC-target. (**G**) The top 10 terms of BPs, CCs, and MFs enrichment analysis from GO enrichment analysis. (**H**) KEGG enrichment analysis of potential therapeutic targets for galangin against HCC

As shown in Fig. 1A, the chemical structure of galangin was obtained from the TCMSP database. Through research of the BATMAN-TCM database and the SwissTargetPrediction website, 120 potential targets of galangin were identified. HCC disease targets were collected from various databases, including 1981 in the GeneCards database, 507 in the OMIM database, 1396 in the DisGeNET database, and 284 in the PharmGKB database. After merging and deleting duplicate targets, a total of 3319 HCC targets were collected (Fig. 1B). By intersecting 120 targets of galangin with 3319 targets of HCC disease, 67 shared targets of galangin against HCC were determined (Fig. 1C).

The target of galangin against HCC was inputted into the STRING database for processing, and a PPI network diagram was obtained (Fig. 1D). The relevant data was imported into Cytoscape 3.9.1 software, and network topology analysis was performed in the software to screen the top ten core targets with the highest degree of centrality (Fig. 1E). These targets include SRC, ESR1, MMP9, CDK4, CCNB1, MMP2, CDK2, CDK1, CHK1 (CHEK1), and PLK1.

67 common targets of galangin and HCC were analyzed for GO and KEGG enrichment. GO functional enrichment analysis showed that the target proteins were involved in 55 biological processes (BPs), 44 cellular components (CCs), and 41 molecular functions (MFs). The top 10 terms, according to gene counts in each category, are presented as column charts (Fig. 1G). The KEGG enrichment analysis showed that galangin can mainly affect 37 signaling pathways, including the cell cycle, pathways in cancer, the PI3K-Akt signaling pathway, microRNAs in cancer, and chemical carcinogenesis - receptor activation. The top 20 pathways enriched by KEGG are shown in Fig. 1H.

The “galangin-target-pathway-HCC” interaction network (Fig. 1F) was constructed by importing information files of galangin, common targets, signaling pathways, and HCC into Cytoscape 3.9.1. The network is composed of 89 nodes and 322 edges. The yellow circle represents the target, the blue diamond indicates the signaling pathway, the red inverted triangle denotes galangin, the green triangle represents HCC, and the lines represent the interconnections between them. As shown in Fig. 1F, the key targets of galangin therapy for HCC, including CCNB1, CDK4, CDK1, and PLK1, belong to the cell cycle signaling pathway (hsa04110).

### Molecular docking analysis of galangin

**Fig. 2:**
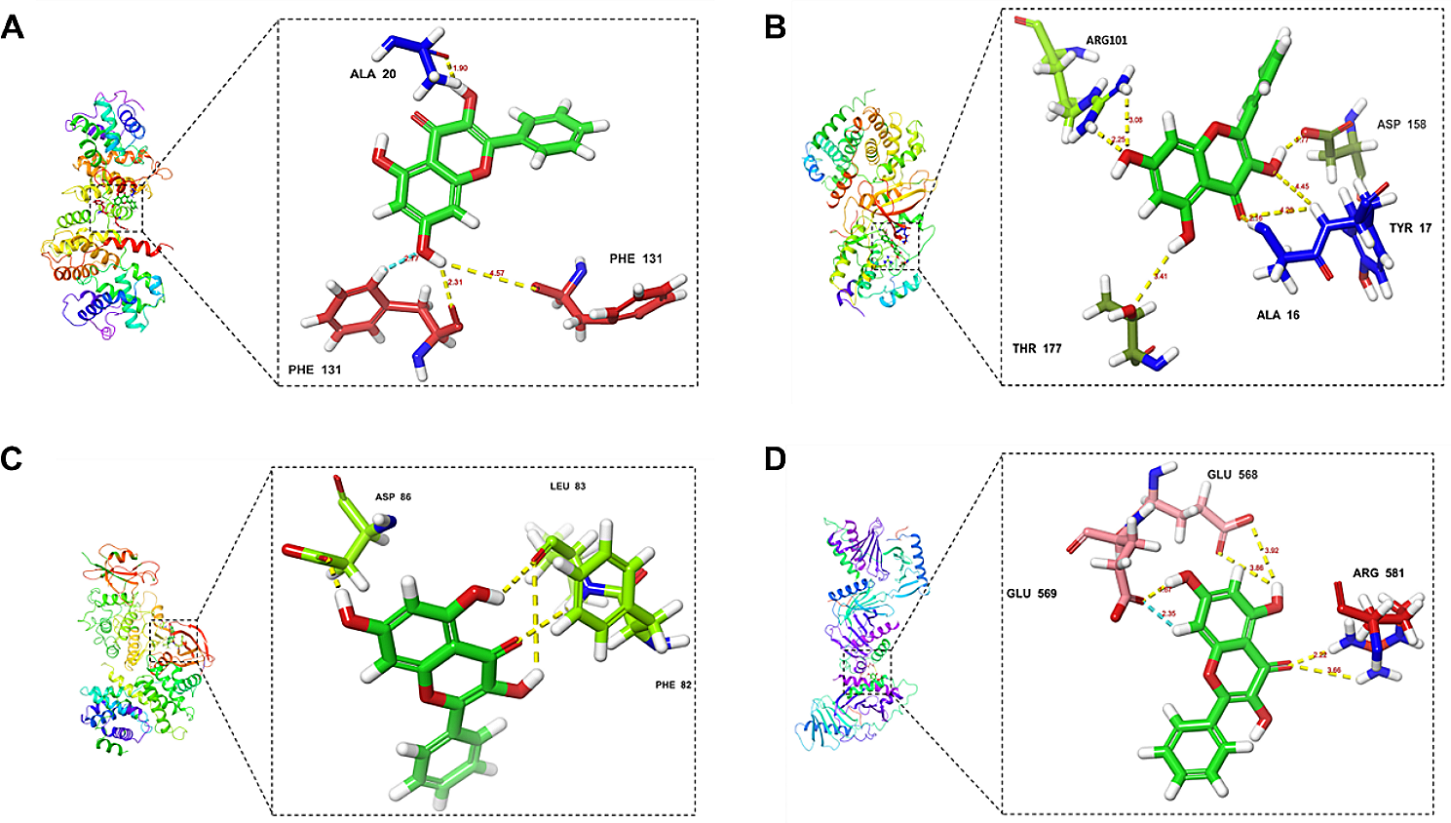
Validation of the interactions between galangin and HCC. (**A**) The docking sites of CCNB1 and galangin. (**B**) The docking sites of CDK4 and galangin. (**C**) The docking sites of CDK1 and galangin. (**D**) The docking sites of PLK1 and galangin

When the absolute value of the binding energy between the ligand and the receptor is greater, the binding is more stable. A binding energy of less than -5 kcal/mol implies a strong affinity between the ligand and the receptor [10]. Docking experiments were conducted using galangin as a ligand with four key targets, including CCNB1, CDK4, CDK1, and PLK1, as receptors. As shown in Fig. 2, the docking results of galangin and its key targets were visualized using PyMOL software. The binding energies of galangin with CCNB1, CDK4, CDK1, and PLK1 were -5.4 kcal/mol, -5.5 kcal/mol, -7.8 kcal/mol, and -5.6 kcal/mol, respectively. The findings indicate that galangin has a good binding affinity with each of these key receptors.

### Galangin suppressed the viability and proliferation of HCC cells

**Fig. 3:**
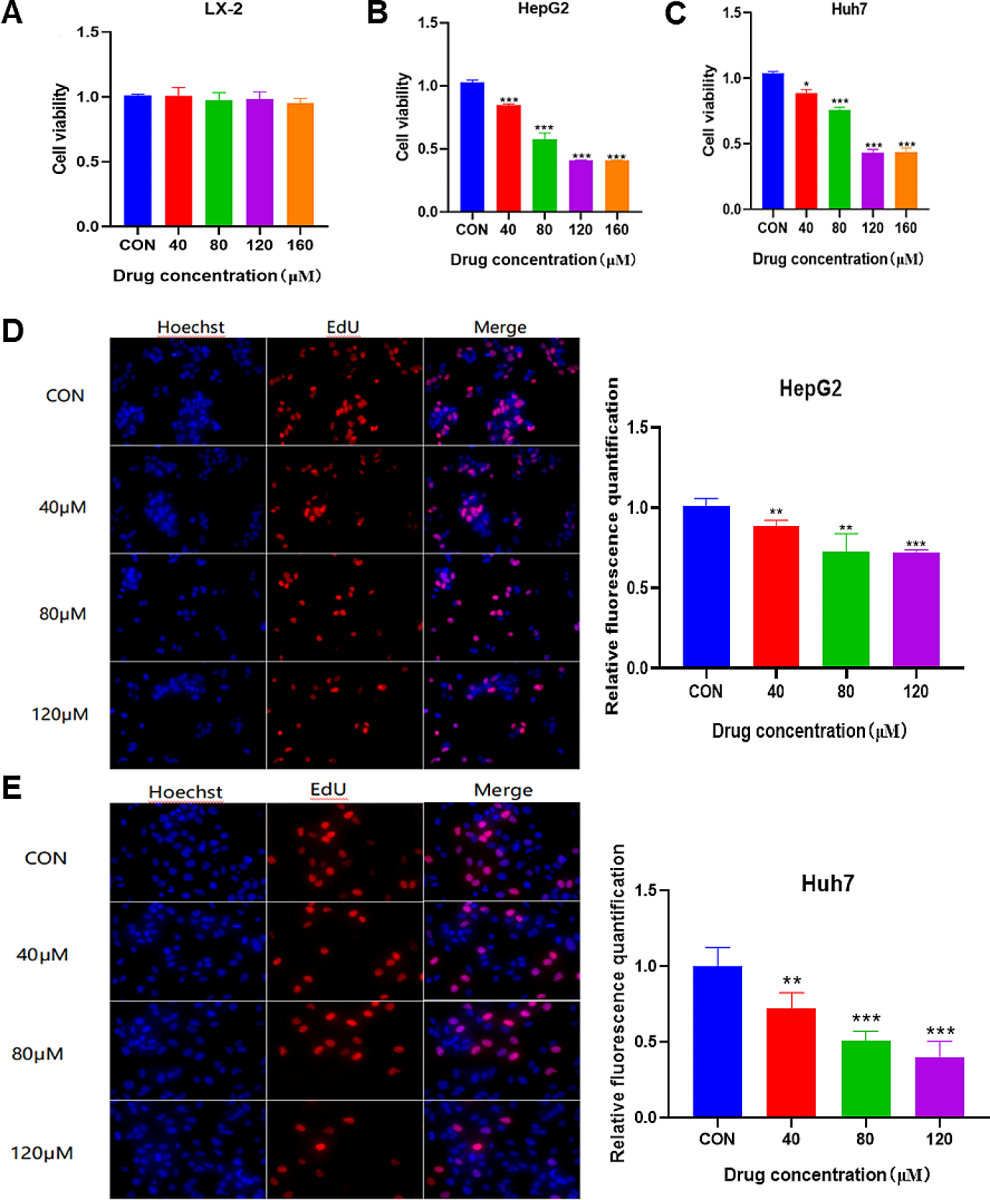
Effect of galangin on viability and proliferation of three cell lines. The cytotoxicity of galangin (40, 80, 120, and 160 µM) on (**A**) LX-2, (**B**) HepG2, and (**C**) Huh7 cells was determined using the CCK8 method. Effect of galangin (40, 80, and 120 µM) on the proliferation of (**D**) HepG2 cells and (**E**) Huh7 cells. ^*^p < 0.05, ^**^p < 0.01, and ^***^p < 0.001 vs. control group

As shown in Fig. 3A, galangin had no inhibitory effect on the proliferation of LX-2 cells at concentrations of 40–160 µM for 12 h (*p* > 0.05). On the contrary, galangin showed a significant inhibitory effect on the proliferation of HepG2 cells (*p* < 0.001) and Huh7 cells (*p* < 0.05) (Fig. 3B and C). The results indicated that galangin displayed cytotoxic effects on HCC cells, including HepG2 and Huh7 cells, within the concentration range of 40–160 µM. Additionally, it impeded the proliferation of these cancer cells while showing no cytotoxicity towards the normal LX-2 cells. The half maximal inhibitory concentration (IC50) of galangin on HepG2 and Huh7 cells was 73.88 µM and 82.41 µM, respectively. Consequently, concentrations of 40, 80, and 120 µM of galangin were chosen for subsequent experiments. In addition, EDU staining experiments confirmed that cell proliferation was attenuated in HepG2 and Huh7 cells treated with galangin (Fig. 3D and E). In summary, these findings support the anti-proliferative effect of galangin on HCC cells in vitro.

### Galangin inhibited cell cycle progression and induced apoptosis in HCC cells

**Fig. 4:**
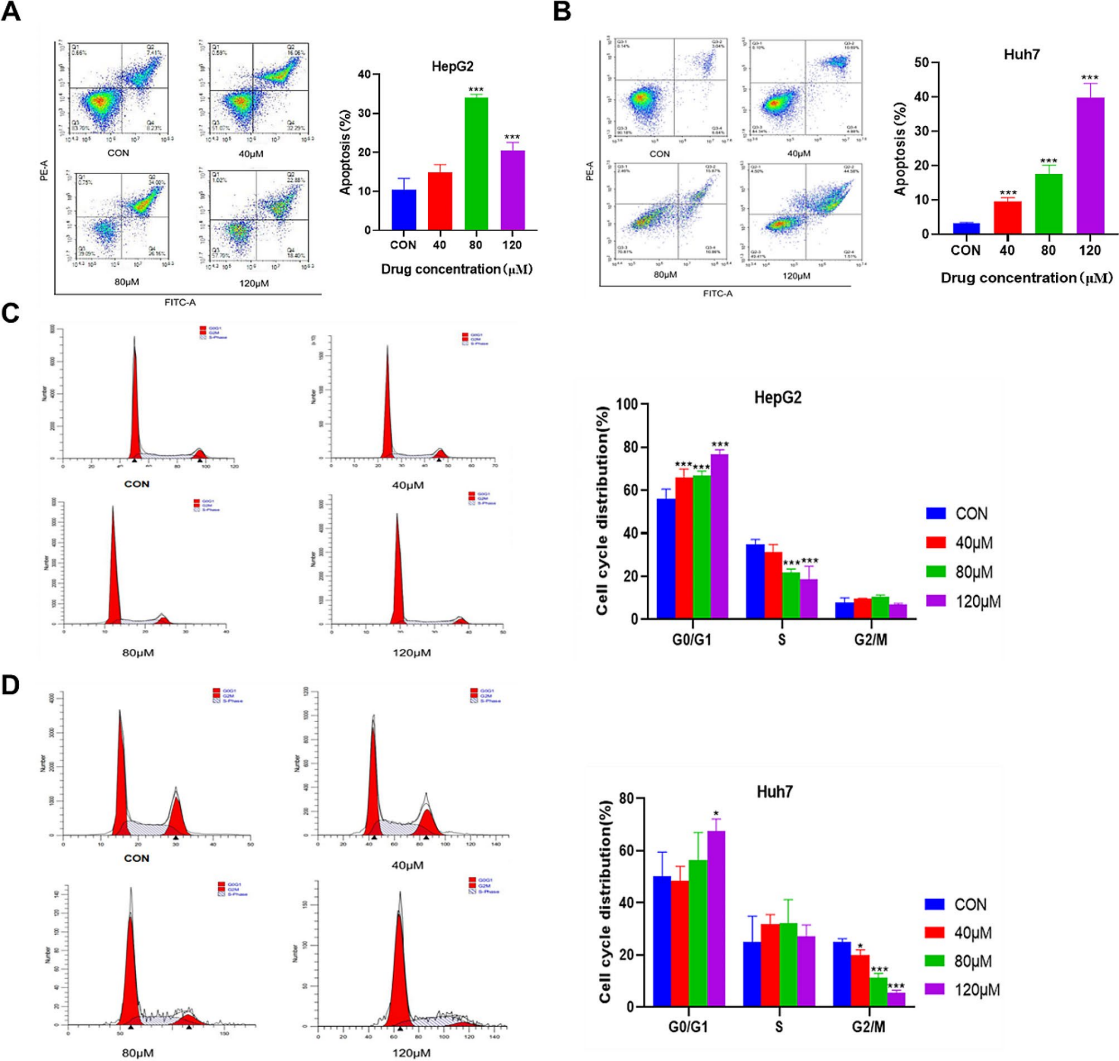
Effects of galangin treatments on HCC cell apoptosis and cell cycle progression. The apoptosis of HepG2 (**A**) and Huh7 cells (**B**) was evaluated using flow cytometry. The cell cycle progression of (**C**) HepG2 and (**D**) Huh7 cells was measured using flow cytometry. ^*^p < 0.05 and ^***^p < 0.001 vs. control group

As shown in Fig. 4A and B, treatment of HepG2 cells with different concentrations of galangin (40, 80, and 120 µM) resulted in a significant increase in the rate of apoptosis. Specifically, 80 µM (*p* < 0.01) and 120 µM (*p* < 0.001) of galangin significantly increased the apoptosis rate of HepG2 cells. Meanwhile, galangin can induce apoptosis in Huh7 cells in a dose-dependent manner (*p* < 0.001). These results indicate that galangin can induce apoptosis in HepG2 and Huh7 cells and inhibit the proliferation of HCC cells.

The effect of galangin on the cell cycle of HepG2 cells was further detected by flow cytometry. HepG2 and Huh7 cells were exposed to different concentrations of galangin (40, 80, and 120 µM) for 12 h. As shown in Fig. 4C, compared to the control group, the percentage of HepG2 cells in the G0/G1 phase significantly increased in the galangin administration group (*p* < 0.001), while the percentage of HepG2 cells in the S phase significantly decreased (*p* < 0.001). Similarly, the percentage of Huh7 cells in the G0/G1 phase significantly increased in the galangin administration groups (*p* < 0.05), while the percentage of Huh7 cells in the G2/M phase significantly decreased (*p* < 0.05) (Fig. 4D). The results of the cell cycle experiment demonstrate that galangin could block cell cycle progression in HepG2 and Huh7 cells and inhibit the proliferation of HCC cells.

**Fig. 5:**
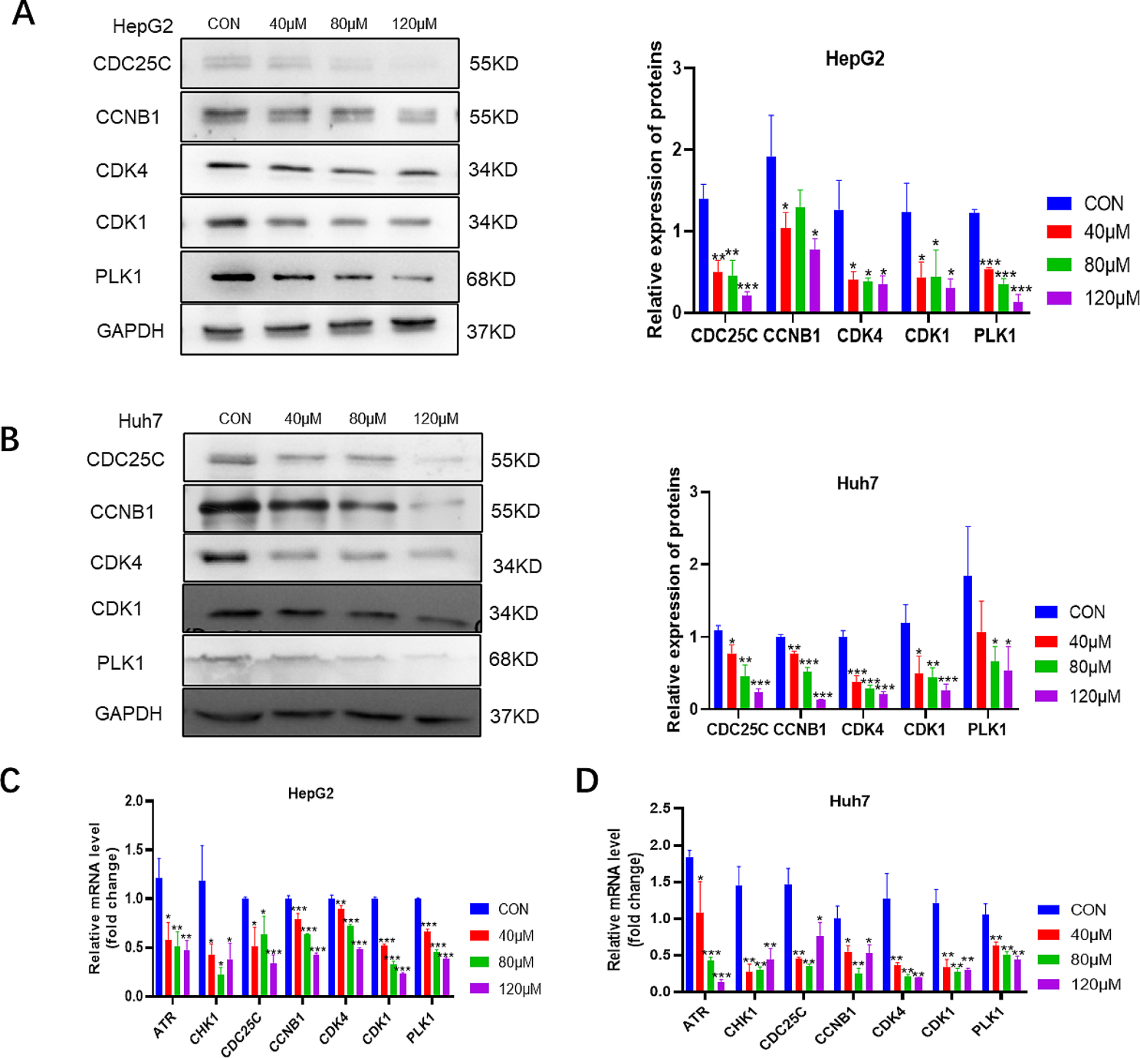
The effect of galangin on the mRNA and protein expression of core targets in HCC cells. Effect of galangin on the expression of CDC25C, CCNB1, CDK4, CDK1, and PLK1 proteins in (**A**) HepG2 and (**B**) Huh7 cells. Effect of galangin on the expression of ATR, CHK1, CDC25C, CCNB1, CDK4, CDK1, and PLK1 mRNA in (**C**) HepG2 and (**D**) Huh7 cells. ^*^p < 0.05, ^**^p < 0.01, and ^***^p < 0.001 vs. control group

### Galangin downregulated the expression of the core targets

As shown in Fig. 5A and B, Western Blot was used to detect the expression of CCNB1, CDK4, CDK1, and PLK1 proteins in HepG2 and Huh7 cells treated with different concentrations of galangin (40, 80, and 120 µM) for 12 h. Compared to the control group, the expressions of CCNB1, CDK4, CDK1, and PLK1 in HepG2 cells were significantly decreased (*p* < 0.05, *p* < 0.01, or *p* < 0.001) in a dose-dependent manner. Similarly, galangin also inhibited the expression of CCNB1, CDK4, CDK1, and PLK1 in Huh7 cells in a dose-dependent manner (*p* < 0.05, *p* < 0.01, or *p* < 0.001). CDC25C, a dual-specificity phosphatase, is essential for controlling the serine/threonine kinases involved in the cell cycle. Our experiments found that galangin significantly downregulated the expression level of the CDC25C protein in HepG2 and Huh7 cells.

In order to further study the effect of galangin on cell cycle-related targets, qRT-PCR was used to detect the mRNA expression of different concentrations of galangin on the relevant targets. As shown in Fig. 5C and D, the mRNA expressions of ATR, CHK1, CDC25C, CCNB1, CDK4, CDK1, and PLK1 were significantly downregulated in HepG2 and Huh7 cells treated with different concentrations of galangin. These results further corroborated that galangin is capable of modulating the core targets of the cell cycle by suppressing the ATR/CHK1 DNA damage response pathway, thereby exhibiting anti-HCC activity.

### The differential expression of core targets in HCC was examined through bioinformatics analysis

**Fig. 6:**
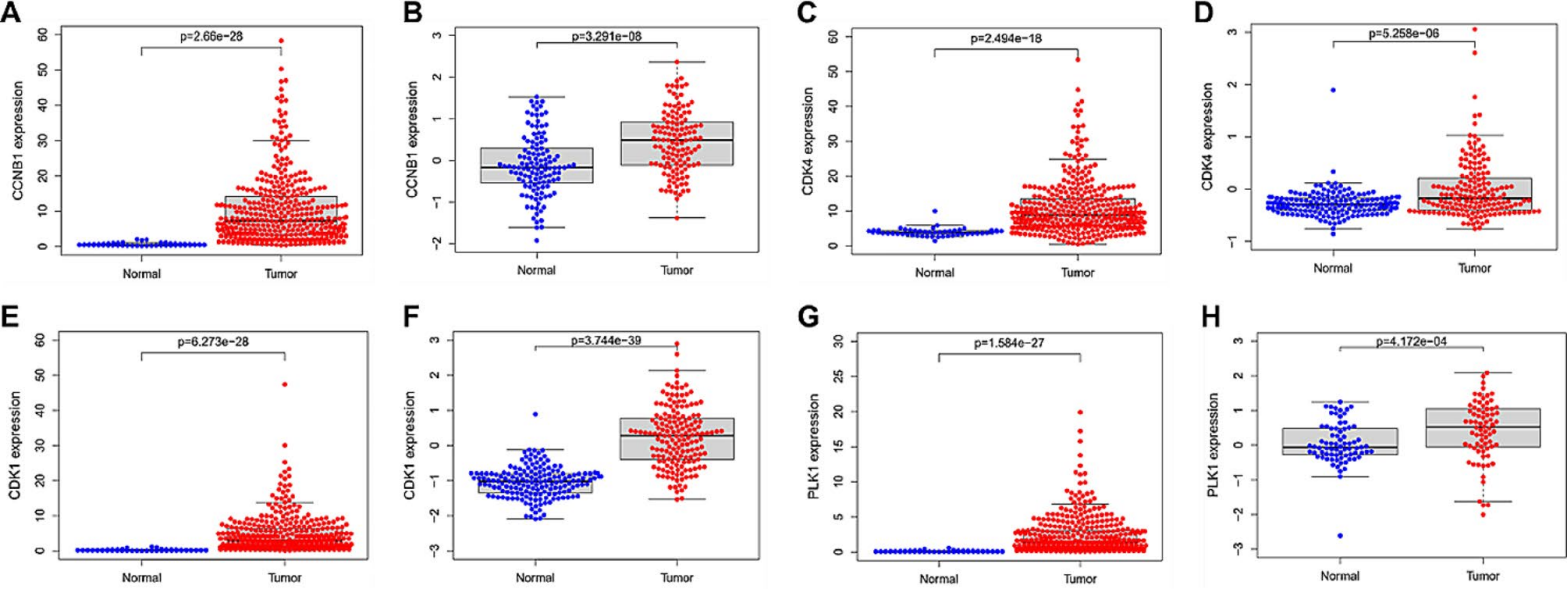
Differential expression of core targets in liver tissue. (**A**) Differential expression of CCNB1 mRNA in the liver. (**B**) Differential expression of CCNB1 protein in the liver. (**C**) Differential expression of CDK4 mRNA in the liver. (**D**) Differential expression of CDK4 protein in the liver. (**E**) Differential expression of CDK1 mRNA in the liver. (**F**) Differential expression of CDK1 protein in the liver. (**G**) Differential expression of PLK1 mRNA in the liver. (**H**) Differential expression of PLK1 protein in the liver

In this study, the mRNA expressions of CCNB1, CDK4, CDK1, and PLK1 in liver tissue were analyzed using the TCGA database. As shown in Fig. 6A, C, E, and G, the mRNA expression levels of CCNB1 (*P* = 2.66e-28), CDK4 (*P* = 2.494e-18), CDK1 (*P* = 6.273e-28), and PLK1 (*P* = 1.584e-27) in the HCC group were significantly higher than those in the normal group. To further clarify the expression of CCNB1, CDK4, CDK1, and PLK1 at the protein level in the liver, high-throughput proteomic data based on mass spectrometry technology was analyzed in the CPTAC database. As shown in Fig. 6B, D, F, and H, compared to the normal group, the expressions of CCNB1 (*P* = 3.291E-08), CDK4 (*P* = 5.258E-06), CDK1 (*P* = 3.744E-39), and PLK1 (*P* = 4.172E-04) proteins were upregulated in HCC patients. The results above indicate that CCNB1, CDK4, CDK1, and PLK1 are upregulated in the livers of patients with HCC at both the mRNA and the protein levels.

**Fig. 7:**
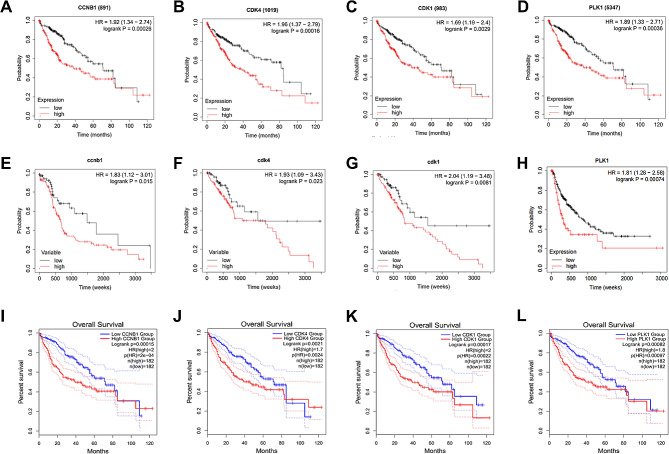
The relationship between core target expression and overall survival in HCC patients. The effect of CCNB1 (**A**), CDK4 (**B**), CDK1 (**C**), and PLK1 (**D**) mRNA expression levels on the survival of HCC patients was evaluated using the TCGA database. The effect of CCNB1 (**E**), CDK4 (**F**), CDK1 (**G**), and PLK1 (**H**) protein expression levels on the survival of HCC patients was evaluated using the CPTAC database. The effect of CCNB1 (**I**), CDK4 (**J**), CDK1 (**K**), and PLK1 (**L**) mRNA expression levels on the survival of HCC patients was further confirmed by the Kaplan-Meier plotter online database

The effects of mRNA expression of CCNB1, CDK4, CDK1, and PLK1 on the survival of HCC patients were evaluated using the TCGA database. The median mRNA expression levels of CCNB1, CDK4, CDK1, and PLK1 were used as cut-off values to divide HCC patients into high-expression and low-expression groups. Kaplan-Meier survival analysis showed that the overall survival rate of HCC patients in the high mRNA expression group of CCNB1 (*P* = 0.00026), CDK4 (*P* = 0.00016), CDK1 (*P* = 0.0029), and PLK1 (*P* = 0.00036) was significantly lower than that of those in the low mRNA expression group (Fig. 7A-D). Furthermore, the relationship between the expression levels of CCNB1, CDK4, CDK1, and PLK1 proteins in the liver and the survival of HCC patients was validated using the CPTAC database. Compared to the low-expression group of HCC patients, those with high expression levels of CCNB1 (*P* = 0.015), CDK4 (*P* = 0.023), CDK1 (*P* = 0.00081), and PLK1 (*P* = 0.00074) proteins had a lower overall survival rate (Fig. 7E-H). The Kaplan-Meier Plotter online database was utilized to ascertain the correlation between the mRNA expression levels of CCNB1, CDK4, CDK1, and PLK1 and the survival rate of HCC patients. The results showed that the mRNA expression levels of CCNB1 (*P* = 0.00015), CDK4 (*P* = 0.0021), CDK1 (*P* = 0.00017), and PLK1 (*P* = 0.00082) were negatively correlated with the overall survival rate of HCC patients (Fig. 7I-L). These findings are consistent with the analysis results obtained from the TCGA database. Therefore, the results of bioinformatics analysis demonstrate that CCNB1, CDK4, CDK1, and PLK1 are key targets for HCC therapy.

## Discussion

HCC is a common primary liver malignancy. The incidence of HCC is increasing year by year, with a high mortality rate and a poor prognosis. With the advancement of research in traditional Chinese medicine, it is of great scientific and practical value to extract effective and less toxic active ingredients and reveal their anticancer mechanisms. Galangin is the main functional component extracted from *Alpinia officinarum* Hance [11, 12]. Many studies have shown that galangin can inhibit the growth and invasion of various malignant tumors, including lung cancer [13], breast cancer [14], liver cancer [15], bladder cancer [16], and leukemia [17]. Its anticancer spectrum is relatively wide and has a good application prospect. Although galangin has been extensively studied as an antineoplastic agent in various cancers, its mechanism of action in HCC remains unclear.

The employment of network pharmacology introduces a novel methodology to investigate the mechanisms of drug therapy for diverse diseases. In this study, the results of network pharmacology showed that the key targets of galangin against liver cancer were SRC, ESR1, MMP9, CDK4, CCNB1, MMP2, CDK2, CDK1, CHEK1, and PLK1. Among these, CCNB1, CDK1, CDK4, and PLK1 are important regulators of the cell cycle. Pathway enrichment analysis also revealed that the cell cycle pathway is a crucial pathway for galangin therapy in HCC. The cell-cycle signaling pathway is a common activation pathway in cancer development, which can regulate tumor cell apoptosis and the cell cycle [18]. As a member of the cyclin family, CCNB1 is highly expressed in a variety of human tumor tissues and is closely related to tumor cell proliferation, metastasis, and poor prognosis in patients [19]. CDK1 is an important regulatory protein in the cell cycle and is the only member of the CDK family that can independently regulate the cell cycle [20]. Studies have shown that CCNB1 and CDK1 are highly expressed in patients with HCC. The siRNA-mediated knockdown of the CCNB1 and CDK1 genes significantly induces autophagy and senescence in HCC cells [19]. The co-administration of CCNB1 and CDK1 inhibitors with immunotherapy has been shown to increase the survival time of patients with HCC [21]. CDK4 is the first cyclin-dependent protein kinase activated during the cell proliferation stage and is a rate-limiting factor of the G1/S phase transition in the cell cycle. The proliferation of human HCC cell lines is inhibited by CDK4 inhibitors through the promotion of reversible cell cycle arrest [22]. PLK1 is an important kinase that regulates the cell cycle and is abundantly expressed in HepG2 cells. Studies have shown that knocking down the PLK1 gene can significantly inhibit the migration of human liver cancer vascular endothelial cells [22, 23]. In this study, bioinformatics analysis found that HCC patients with high expression of CCNB1, CDK4, CDK1, and PLK1 proteins and mRNA had a lower overall survival rate. In vitro tests showed that the protein and mRNA expression levels of CCNB1, CDK4, CDK1, and PLK1 in HepG2 and Huh7 cells were significantly reduced after exposure to various concentrations of galangin. In addition, the molecular docking results demonstrated the robust binding affinity of galangin with these targets. Taken together, these results suggest that galangin may induce apoptosis in liver cancer cells by disrupting the cell cycle through its influence on cell cycle proteins such as CCNB1, CDK4, CDK1, and PLK1.

**Fig. 8:**
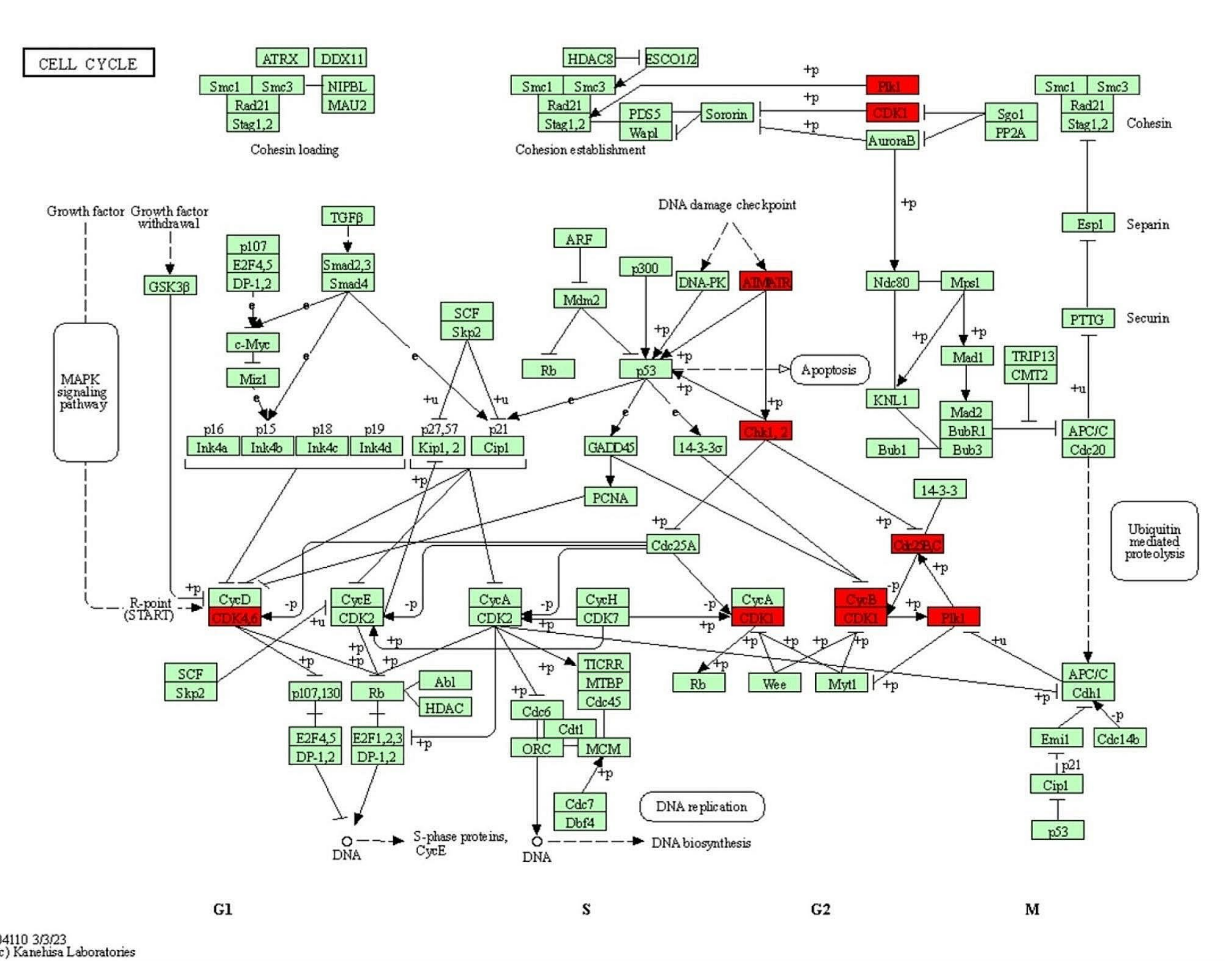
The cell cycle signaling pathway interfered with by galangin. The red rectangles represent potential targets for galangin

During evolution, cells have developed a rigorous and effective set of checkpoint mechanisms to ensure that their genetic material is accurately replicated and transmitted to subsequent generations [24]. DNA damage activates the cell cycle checkpoints, which stop the cell cycle, giving the DNA enough time to repair, or causing the cell to die if the damage is irreparable. The regulation of the cell cycle from the end of the G1 phase to the S phase and G2 phase is predominantly controlled by the ATR/CHK1/CDC25 pathway. After DNA damage, ATR accumulates at the damaged site and becomes activated. Its downstream target, CHK1, is subsequently activated, which then activates p53. This ultimately leads to cell cycle arrest. Cell division cycle 25 C (CDC25C) is crucial for regulating the activity of serine/threonine kinases involved in the cell cycle. Moreover, CDC25C can activate the CCNB1/CDK1 complex by triggering dephosphorylation of CDK1, thereby promoting the G2/M transition in mitotic cells. In the present work, the expression of ATR, CHK1, and CDC25C mRNA was detected in HepG2 and Huh7 cells treated with galangin. The results showed that galangin effectively reduced the expression of ATR, CHK1, and CDC25C mRNA in HCC cells. The findings of the cell cycle studies showed that galangin inhibited the proliferation of HCC cells in the G0/G1 phase. The cell cycle signaling pathway interfered with by galangin is shown in Fig. 8.

In this study, network pharmacology was used to predict the targets of galangin in the treatment of HCC, which were subsequently validated through in vitro experiments. Therefore, it is imperative for future research to prioritize conducting experimental validation in animal models to offer more precise evidence supporting the efficacy of galangin in treating HCC. Although molecular docking simulations can unveil the interactions between galangin and its targets, they are inadequate in fully representing its pharmacological and biological effects in the human body. Consequently, it is crucial to carry out in-depth studies on the treatment process, adverse effects, and prognosis in clinical trials. Such investigations will expedite the utilization of galangin as a therapeutic agent for liver cancer in clinical practice.

## Conclusions

In summary, galangin induces apoptosis in HCC cells by blocking the cell cycle at the G0/G1 phase through the inhibition of key mRNA and protein expression in the cell cycle. The present study provides a new research direction for the treatment of HCC using galangin and offers ideas and a theoretical basis for investigating lead compounds for the treatment of HCC.

### Electronic supplementary material

Below is the link to the electronic supplementary material.

Supplementary Material 1

## Data Availability

The original contributions presented in the study are included in the article, and further inquiries can be directed to the corresponding author.

## Abbreviations

HCC: Hepatocellular carcinoma
qPCR: Quantitative real-time PCR
BP: Biological processes
CC: Cellular components
MF: Molecular functions
CDC25C: Cell division cycle-25 C
GO: Gene Ontology
KEGG: Kyoto Encyclopedia of Genes and Genomes
CDK1: Cyclin-dependent kinase-1
