# Supplementary material for: Integrating network pharmacology, bioinformatics, and experimental validation to unveil the molecular targets and mechanisms of galangin for treating hepatocellular carcinoma
Source: BMC Complement Med Ther. 2024 May 30;24:208. doi: 10.1186/s12906-024-04518-x (PMC11137903; doi:10.1186/s12906-024-04518-x)
Supplement: Supplementary file 1 — Supplementary Material 1 [file 12906_2024_4518_MOESM1_ESM.docx]

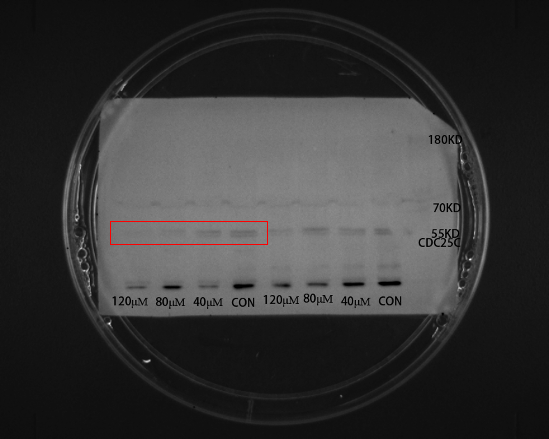


**Supplementary Figure S1.** The effect of galangin on the expression of CDC25C in HepG2 cells.


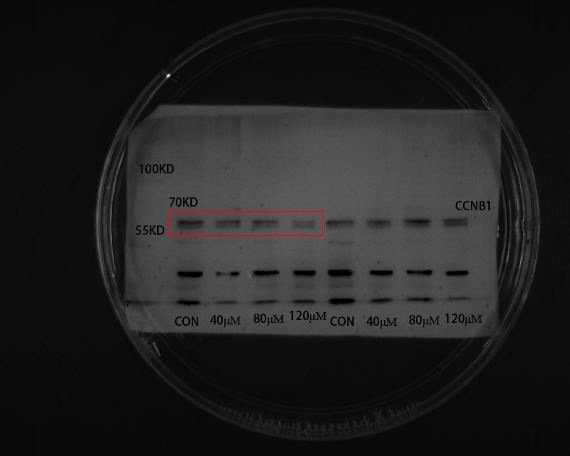


**Supplementary Figure S2.** The effect of galangin on the expression of CCNB1 in HepG2 cells.


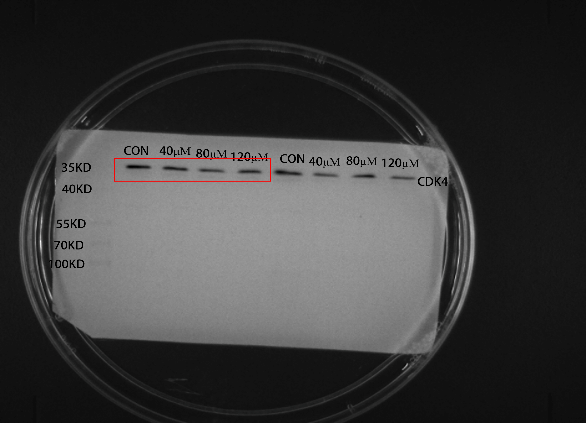


**Supplementary Figure S3.** The effect of galangin on the expression of CDK4 in HepG2 cells.


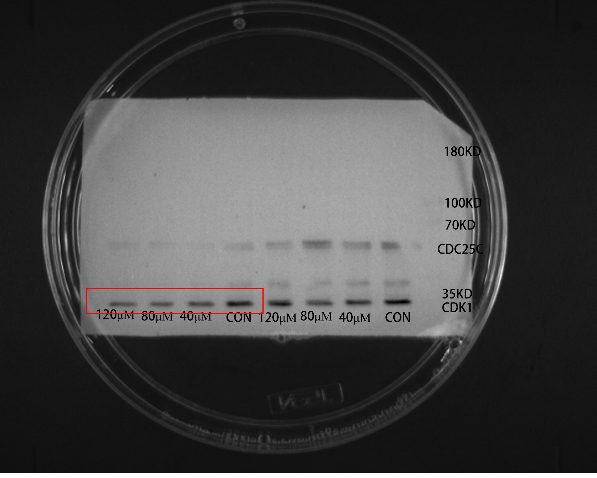


**Supplementary Figure S4.** The effect of galangin on the expression of CDK1 in HepG2 cells.


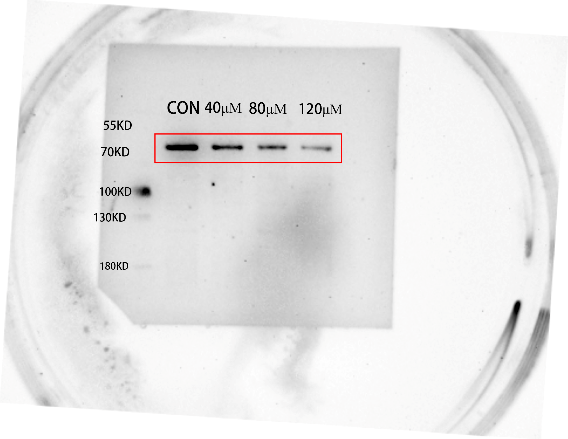


**Supplementary Figure S5.** The effect of galangin on the expression of PLK1 in HepG2 cells.


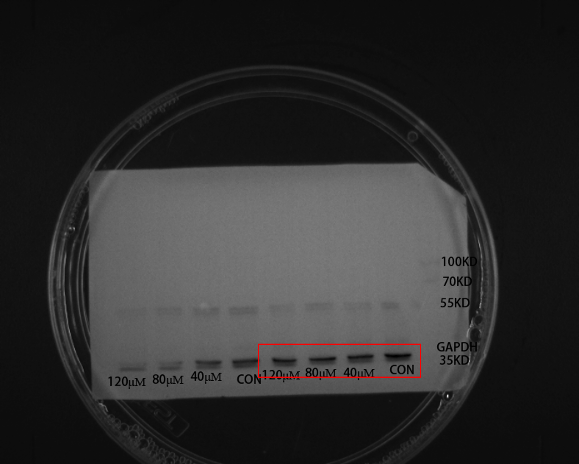


**Supplementary Figure S6.** The effect of galangin on the expression of GAPDH in HepG2 cells.


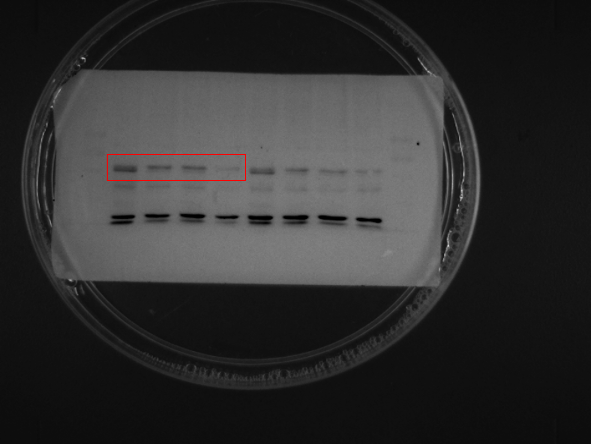


**Supplementary Figure S7.** The effect of galangin on the expression of CDC25C in Huh7 cells.


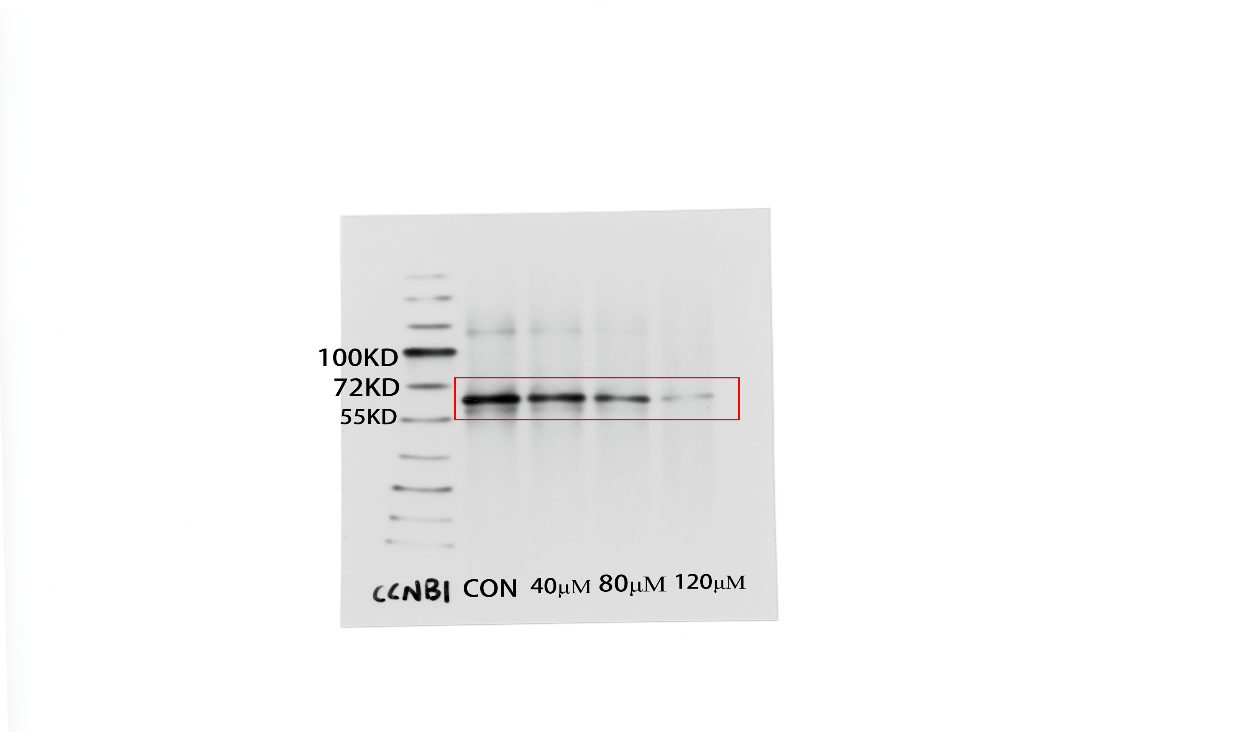


**Supplementary Figure S8.** The effect of galangin on the expression of CCNB1 in Huh7 cells.


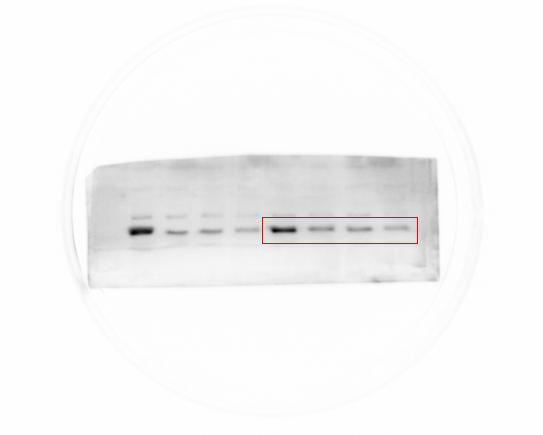


**Supplementary Figure S9.** The effect of galangin on the expression of CDK4 in Huh7 cells.


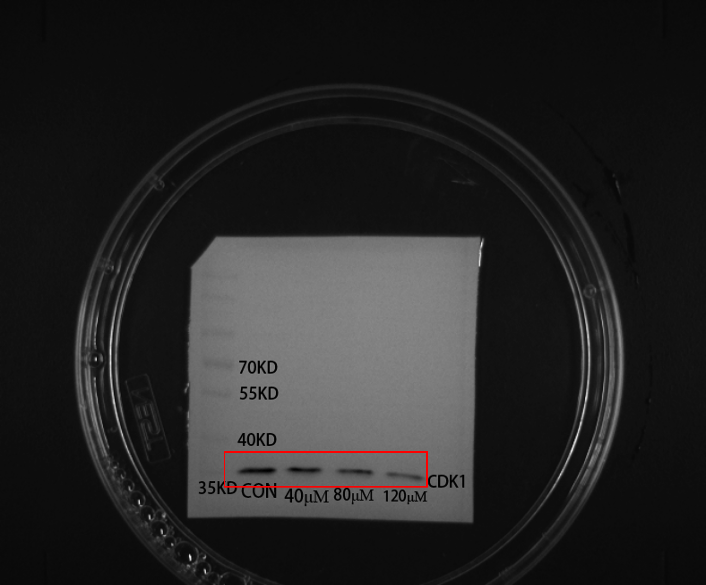


**Supplementary Figure S10.** The effect of galangin on the expression of CDK1 in Huh7 cells.


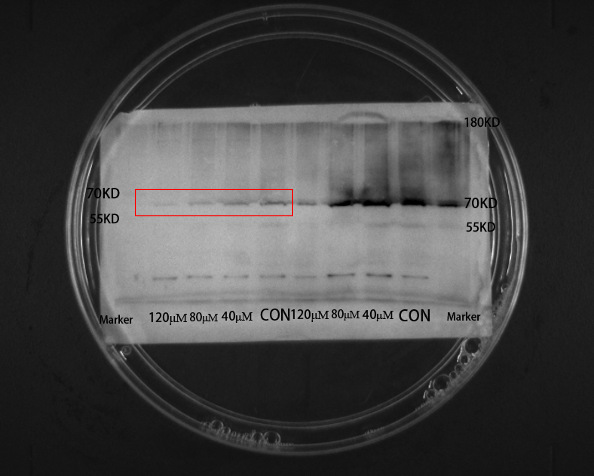


**Supplementary Figure S11.** The effect of galangin on the expression of PLK1 in Huh7 cells.


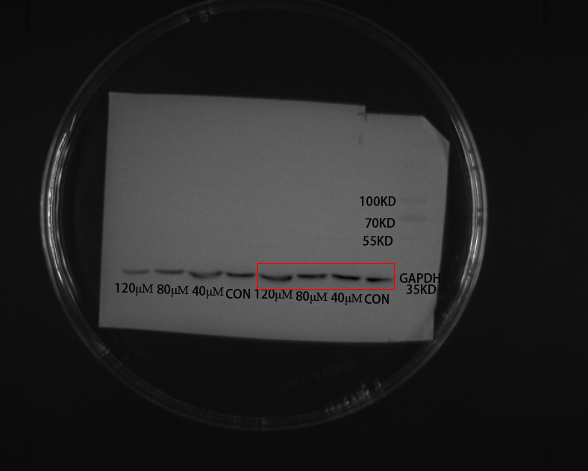


**Supplementary Figure S12.** The effect of galangin on the expression of GAPDH in Huh7 cells.
